# Antimicrobial Efficacy and Biocompatibility of a Denture Cleanser Containing *Paeonia lactiflora* Extract

**DOI:** 10.3390/biomedicines13040869

**Published:** 2025-04-03

**Authors:** Ji-Won Lim, Jiyeon Lee, Min-Kyung Kang, Hee-Eun Kim

**Affiliations:** 1 Department of Health Science, Graduate School, Gachon University, Yeonsu-gu, Incheon 21936, Republic of Korea; jiwon219@gachon.ac.kr (J.-W.L.); angel3327@gachon.ac.kr (J.L.); 2 Department of Dental Hygiene, Hanseo University, Seosan 31963, Chungcheongnam-do, Republic of Korea; 3 Department of Dental Hygiene, Gachon University College of Medical Science, Yeonsu-gu, Incheon 21936, Republic of Korea

**Keywords:** antimicrobial effects, biocompatibility, denture cleanser, oral biofilm, *Paeonia lactiflora*, red fluorescence

## Abstract

**Background/Objectives**: Microbial biofilms on denture surfaces pose significant oral and systemic health risks. Although chemical denture cleansers are widely used, they can cause mucosal irritation and disrupt the oral microbiome. The aim of this study was to evaluate the antimicrobial efficacy and biocompatibility of a denture cleanser containing *Paeonia lactiflora* extract (DC-PL) as a potential natural alternative. **Methods**: Oral microcosm biofilms were formed using human saliva and matured over 6 days. Then, the biofilms were treated for 1 min daily over 6 days with DC-PL, distilled water (DW; negative control), or Polident^®^ (PD; positive control). Antimicrobial effects were assessed by measuring the red fluorescence intensity (ratio of red to green fluorescence intensity [Ratio_R/G_]) and aciduric bacterial counts. Biocompatibility was evaluated through an oral mucosal irritation test. A one-way analysis of variance followed by Tukey’s post hoc test was used for between-group comparisons. **Results**: Ratio_R/G_ in the DC-PL group was significantly lower than that in the DW group (0.94-fold, *p* = 0.021) and comparable with that in the PD group (*p* = 0.502). Aciduric bacterial counts in the DC-PL group were 0.92-fold lower than those in the DW group (*p* = 0.037) and comparable with those in the PD group (*p* = 0.460). The oral mucosal irritation index was 0, indicating no irritation. **Conclusions**: DC-PL demonstrated antimicrobial efficacy similar to that of PD while maintaining excellent biocompatibility. These findings underscore its potential as a safe and effective alternative to conventional chemical cleansers, offering a clinically viable solution for improving oral health management.

## 1. Introduction

Biofilms are complex, structured microbial communities composed of diverse bacteria and fungi embedded in a self-produced matrix of extracellular polymeric substances, firmly adhering to living or inert surfaces [1]. Microbial biofilms that develop on denture surfaces pose a persistent challenge for individuals relying on removable prostheses [2]. These biofilms not only compromise oral hygiene but also contribute to a range of health complications [3]. The susceptibility of dentures to biofilm formation is largely influenced by the properties of the materials used and fit-related factors [4]. The inherent porosity and surface roughness of denture base materials create an ideal environment for bacterial and fungal adhesion [5], while poor adaptation to the oral mucosa can impair salivary flow and hinder natural cleansing mechanisms [6]. Consequently, denture-related biofilms are closely associated with an increased risk of oral mucosal inflammation, denture stomatitis, and even the dissemination of pathogenic microorganisms throughout the oral cavity and beyond [7]. Notably, these biofilms can serve as reservoirs for respiratory pathogens, potentially increasing the risk of systemic conditions such as pneumonia, cardiovascular diseases, and diabetes through inflammatory and immune-mediated pathways [8,9]. A recent study also suggested a potential link between chronic denture biofilm exposure and neurodegenerative diseases, including Alzheimer’s disease, due to systemic inflammation and bacterial translocation into the bloodstream [10]. Therefore, the effective management of denture biofilms is critical for not only maintaining oral health but also preventing broader systemic complications [11].

Conventional denture cleaning methods, including mechanical and chemical approaches, have long been used to control microbial biofilms [12]. While brushing can remove surface biofilms, it is often ineffective in eliminating microbial deposits in hard-to-access areas. Moreover, repeated friction can cause microdamage to denture materials, compromising their longevity [13]. Chemical denture cleansers, such as effervescent tablets containing oxidizing agents, offer an alternative; however, their use comes with notable drawbacks, including residual toxicity and mucosal irritation [14,15]. In particular, sodium hypochlorite-based cleansers, known for their potent bactericidal properties, can inadvertently disrupt the delicate balance of the oral microbiome, leading to issues such as *Candida albicans* overgrowth and mucosal irritation [16,17]. These limitations highlight the urgent need for safer and more biocompatible solutions that can effectively manage denture biofilms while preserving the ecological balance of the oral microbiota.

To overcome this challenge, natural antimicrobial agents have been introduced as promising alternatives because of their ability to selectively suppress pathogenic microorganisms while maintaining a balanced microbial ecosystem [18,19]. Among these, *Paeonia lactiflora* (PL), a traditional medicinal herb, has attracted significant attention owing to its antioxidant, anti-inflammatory, and antibacterial properties [20,21]. A previous study demonstrated that PL extract effectively inhibits the pathogenicity of oral microcosm biofilms, making it a strong candidate ingredient for denture cleansers [22]. However, while the antimicrobial potential of PL has been established, to the best of our knowledge, to date, no studies have evaluated the efficacy and biocompatibility of denture cleansers formulated with PL extract. Given the risks associated with conventional chemical cleansers, it is crucial to ensure that alternative solutions not only provide strong antimicrobial effects but also meet safety standards for intraoral use [23].

Therefore, the aim of this study was to assess the antimicrobial efficacy and biocompatibility of a denture cleanser containing PL extract (DC-PL) as a potential natural alternative. The null hypothesis tested was that the DC-PL has no substantial antimicrobial effects compared with distilled water (DW) and a conventional chemical cleanser (Polident^®^; PD) and induces marked oral mucosal tissue irritation.

## 2. Materials and Methods

### 2.1. Ethical Considerations and Sample Size Determination

This study was approved by the Gachon University Institutional Review Board (approval number: 1044396-202410-HR-163-01) and the Yonsei University Institutional Animal Care and Use Committee (approval number: 2015-0373). The purpose and procedures of the study were explained in detail to participants, and written informed consent was obtained prior to their participation. Inclusion criteria included good systemic and oral health with no history of smoking or alcohol abuse. Participants with active dental caries, periodontal disease, or a history of antibiotic use within the last 3 months were excluded. Saliva donors were instructed to refrain from all oral hygiene activities, including toothbrushing, for 24 h before saliva collection.

The required sample size was calculated using the G*Power program (version 3.1; Heinrich Heine University Düsseldorf, Düsseldorf, Germany). On the basis of previous studies, the effect size (f) was set to 0.5, the probability of alpha error to 0.05, and the statistical power to 0.80 for comparisons using a one-way analysis of variance (ANOVA) among three groups [22]. The total sample size required was determined to be 42.

### 2.2. Preparation of a DC-PL

Freeze-dried PL extract powder was prepared using a previously described method [24]. The PL powder was mixed with 1% cocamidopropyl betaine solution (CocoBetaina; The Modern Co., Ltd., Bucheon, Republic of Korea), a natural surfactant, to prepare a liquid DC-PL at a final concentration of 100 μg/mL. The solution was stirred at 20–25 °C for 24 h.

### 2.3. Formation of Oral Microcosm Biofilms

For collection of stimulated saliva, donors were instructed to chew paraffin wax (Ivoclar Vivadent, Liechtenstein, Germany) for 3 min. A total of 15 mL of saliva was collected and filtered using sterilized glass wool (Duksan Chemicals, Ansan, Republic of Korea) for the removal of debris. Hydroxyapatite disks (Himed, New York, NY, USA; 7 mm diameter, 2 mm height) were used as a substrate for biofilm formation. The disks were polished using a 400-grit polishing machine (M-PREP 5TM; Allied High Tech Products, Inc., Compton, CA, USA) and positioned at the base of acrylic molds [25]. For the formation of oral microcosm biofilms, each hydroxyapatite disk was placed in a 24-well cell culture plate (SPL Life Sciences, Pocheon, Republic of Korea) and inoculated with 1.5 mL of the filtered saliva. The plates were incubated in a CO_2_ incubator (BB15 CO_2_ incubator, Thermo Fisher Scientific, Waltham, MA, USA) at 37 °C under 10% CO_2_ for 4 h. After incubation, the saliva on the disks was carefully removed, and 1.4 mL of basal medium mucin combined with 0.1 mL of 0.5% sucrose (final pH: 7) was added to each well daily for 6 days [25].

### 2.4. Treatment of Oral Microcosm Biofilms

**Figure 1:**
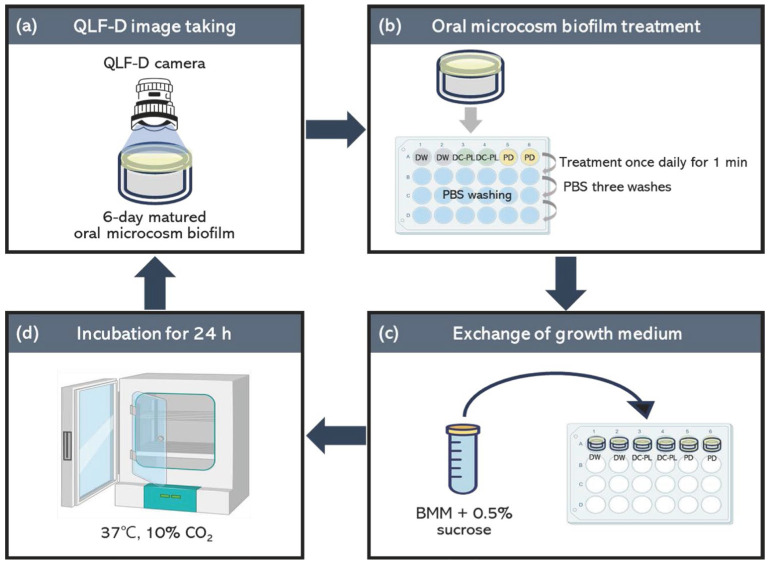
Flowchart of the oral microcosm biofilm treatment process. (**a**) A QLF-D camera was used to capture red fluorescence images of the oral microcosm biofilms prior to any treatment application. (**b**) The samples were immersed for 1 min in one of three test solutions (DW, DC-PL, or PD) and subsequently rinsed three times with phosphate-buffered saline. (**c**) The samples were then nourished with new culture medium consisting of basal medium mucin (1.4 mL) supplemented with 0.1 mL of 0.5% sucrose solution. (**d**) The final step involved transferring the 24-well plate containing the treated samples to an incubation chamber. BMM, basal medium mucin; DW, distilled water; DC-PL, denture cleanser containing PL extract; PBS, phosphate-buffered saline; PD, Polident^®^; QLF-D, quantitative light-induced fluorescence—digital.

The oral microcosm biofilms were treated daily with 1.5 mL of each test solution for 1 min. DC-PL was used in the experimental group, DW served as the negative control, and PD served as the positive control. After treatment, the remaining solutions and debris were removed by rinsing each disk three times with 1.5 mL of phosphate-buffered saline (PBS) (Welgene, Gyengsan, Republic of Korea). Fresh growth medium (1.5 mL) was then added to each well, and the plates were incubated at 37 °C under 10% CO_2_. This process was repeated for 6 consecutive days (Figure 1).

### 2.5. Evaluation of the Antimicrobial Activity of DC-PL

#### 2.5.1. Analysis of Red Fluorescence Intensity

**Figure 2:**
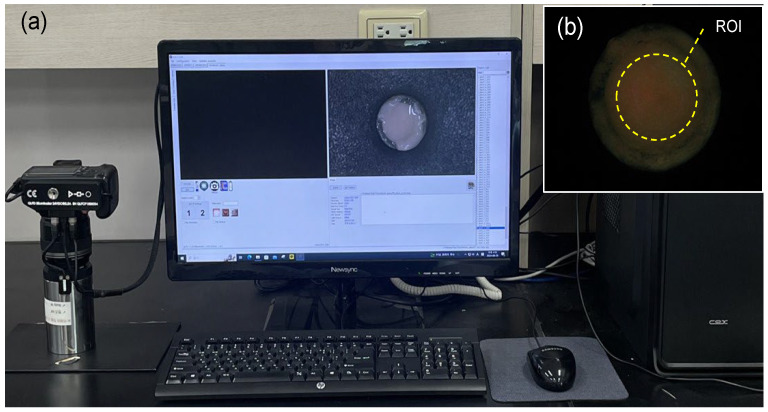
The quantitative light-induced fluorescence-digital (QLF-D) system [25]. The images illustrate (**a**) the QLF-D camera along with the C3 v1.16 software utilized for imaging and (**b**) the designated region of interest (ROI) used to assess red fluorescence intensity within the oral microcosm biofilm. The red fluorescence intensity is quantified by calculating the ratio of red pixels to green pixels in fluorescence images through an image analysis program. ROI, region of interest.

At the end of each treatment session, the oral microcosm biofilms were photographed at 24 ± 1 °C using a quantitative light-induced fluorescence-digital camera (QLF-D; Biluminator™2+; Inspektor Research Systems BV, Amsterdam, The Netherlands) under the blue light mode (Figure 2a). The camera settings were as follows: shutter speed, 1/60 s; aperture value, 7.1; and ISO speed, 1600. A uniform distance of 10 cm was maintained between the camera lens and the sample. The fluorescence images were analyzed using Image PRO version 10 (Media Cybernetics, Inc., Silver Spring, MD, USA). A region of interest (ROI) was consistently defined across all samples based on a previous study [25]. Red and green values within the ROI were measured for each image (Figure 2b), and the ratio of red to green fluorescence intensity (Ratio_R/G_) was calculated to represent oral biofilm pathogenicity. A higher Ratio_R/G_ value was considered to indicate the higher pathogenicity of the oral biofilm [26].

#### 2.5.2. Measurement of Aciduric Bacterial Counts

To measure aciduric bacterial counts in the oral biofilms, brain heart infusion (BHI) agar plates were prepared by adding 37 g of Bacto^TM^ BHI (Becton Dickinson & Co., Franklin Lakes, NJ, USA) and 20 g of Bacto^TM^ agar (Becton Dickinson & Co.) to 1 L of distilled water, with the final pH adjusted to 4.8. After treatment, all disks were rinsed three times with 1.5 mL of PBS to remove any debris, and the biofilm was resuspended in 2 mL of PBS in a 15 mL conical tube (SPL Life Science, Pocheon, Republic of Korea). The biofilm suspensions were homogenized using a vortex mixer (VM-96A; Lab Companion, Seoul, Republic of Korea) and ultrasonic vibrator (Seocho-gu, Seoul, Republic of Korea) for 1 min each. Serial dilutions (10^−1^ to 10^−6^) were prepared, and dilutions from 10^−3^ to 10^−5^ were plated on the BHI agar. The agar plates were incubated at 37 °C under 10% CO_2_ for 72 h, and the aciduric bacterial counts were calculated as the average colony-forming unit count/mL for each plate.

### 2.6. Biocompatibility of DC-PL: Oral Mucosal Irritation Test

Three male Syrian hamsters (8 weeks old, weighing approximately 100–120 g) were purchased from Central Lab Animal Inc (Seocho-gu, Seoul, Republic of Korea). The sample size (n = 3) was determined based on previous studies, ensuring sufficient statistical power for detecting mucosal irritation. Animals with pre-existing oral mucosal injuries, systemic diseases, or abnormal behavior were excluded from the study. No animals were excluded after the experiment began. The experiment was conducted following ISO 10993-10 guidelines [27]. Randomization was not applicable as each hamster served as its internal control, minimizing inter-individual variability. The hamsters were housed individually in cages under controlled conditions (22–24 °C, 12 h light–dark cycle, and 50–60% humidity) with ad libitum access to food and water for 1 week before the study commenced. DC-PL was applied to the right buccal mucosa, which formed the experimental group, while the left buccal mucosa served as the control. DC-PL was applied to an area with a 5 mm diameter for 4 h, with a minimum contact duration of 5 min per hour. The degree of redness at the application site was scored, and the histopathological analysis of excised buccal mucosal tissues was performed by an investigator blinded to the treatment group to minimize observer bias. The tissue samples were evaluated under a fluorescence microscope for four parameters, each scored from 0 to 4 on the basis of severity: epithelial changes (0 = normal; 4 = generalized erosion), leukocyte infiltration (0 = absent; 4 = ≥100 cells), vascular congestion (0 = absent; 4 = marked with vessel disruption), and edema (0 = absent; 4 = marked). The irritation index was calculated as the difference in mean histopathological grades between the experimental and control groups, and the resulting values were used to classify the degree of irritation as follows: 0, none; 1–4, minimal; 5–8, mild; 9–11, moderate; and 12–16, severe.

### 2.7. Statistical Analysis

All data were analyzed using IBM SPSS Statistics version 28.0 (IBM Corp., Armonk, NY, USA), with the significance level set to 0.05. The Kolmogorov–Smirnov test was performed to assess the normality of red fluorescence intensity and aciduric bacterial counts. A one-way ANOVA was performed to compare the effects of the treatments, followed by Tukey’s post hoc test for intergroup comparisons.

## 3. Results

### 3.1. Red Fluorescence Intensity

**Figure 3:**
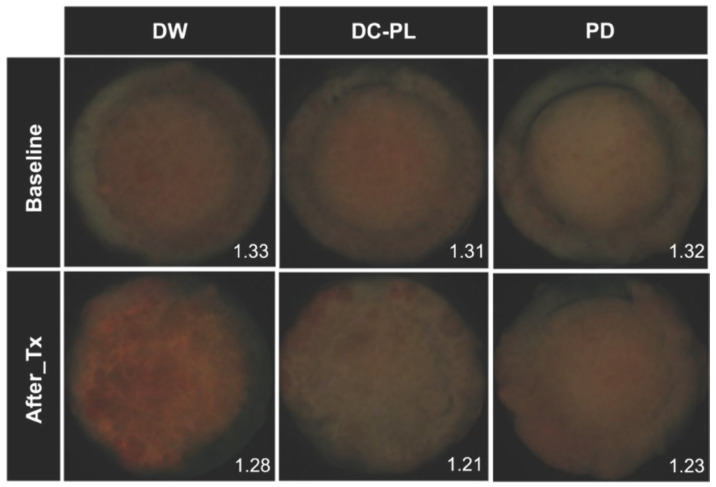
The red fluorescence intensity (Ratio_R/G_) of the oral microcosm biofilms before and after treatment with different agents. The values shown at the bottom right of each image indicate the Ratio_R/G_ of the oral microcosm biofilm. DW, distilled water; DC-PL, denture cleanser containing *Paeonia lactiflora* extract; PD, Polident^®^; Ratio_R/G_, ratio of red to green fluorescence intensity.

The Ratio_R/G_ was 0.94-fold lower in the DC-PL group than in the DW group, representing a significant difference (*p* = 0.021, Table 1). However, no significant difference was observed between the DC-PL and PD groups (*p* = 0.502, Table 1). Fluorescence imaging further confirmed these findings, showing a stronger red fluorescence signal in the DW group than in the DC-PL and PD groups (Figure 3).

### 3.2. Aciduric Bacterial Counts

The aciduric bacterial count in the oral microcosm biofilms was 0.92-fold lower in the DC-PL group than in the DW group, with a significant between-group difference (*p* = 0.037, Table 2). However, no significant differences were observed between the DC-PL and PD groups (*p* = 0.460, Table 2).

### 3.3. Oral Mucosal Irritation

**Figure 4:**
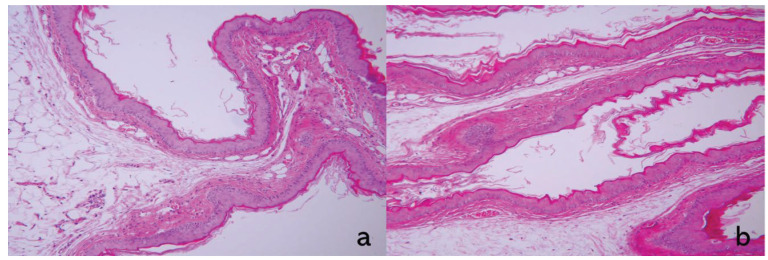
Histopathological evaluation (20× magnification) of oral mucosal irritation. Both the control (**a**) and experimental (**b**) groups exhibit an intact epithelial structure without signs of leukocyte infiltration, vascular congestion, or edema.

The mean histopathological scores for the control and DC-PL groups were 1.7 and 1.3, respectively, resulting in an irritation index of 0, which indicated no oral mucosal irritation (Table 3). Histopathological images showed no notable differences between the experimental and control groups (Figure 4).

## 4. Discussion

In this study, we assessed the antimicrobial efficacy of DC-PL by evaluating red fluorescence intensity (Ratio_R/G_) and aciduric bacterial counts using an oral microcosm biofilm model. We also evaluated its biocompatibility through an oral mucosal irritation test. Our findings demonstrated that DC-PL significantly reduced the red fluorescence intensity of oral biofilms to a level comparable with that achieved by PD, suggesting its inhibitory effect on the biofilm pathogenicity (Table 1). This trend was further corroborated by fluorescence imaging, which showed that the DC-PL and PD groups exhibited weaker red fluorescence than the DW group (Figure 3). These findings align with those of a previous study that investigated the antimicrobial efficacy of denture cleansers containing geraniol and thymol—natural compounds with antibacterial properties similar to PL—against multispecies oral biofilms [28]. Notably, while the denture cleansers in the previous study required extended treatment durations ranging from 5 min to 6 h to achieve antimicrobial efficacy, DC-PL demonstrated effects comparable to those of PD with only 1 min treatment. This suggests that DC-PL may offer a more efficient and time-effective alternative for clinical use. Our findings were also consistent with those of a previous study that evaluated the antimicrobial efficacy of PD using a multispecies biofilm model [29]. However, the multispecies biofilm model used in that study comprised only six bacterial species, whereas our study employed a microcosm biofilm model derived from human saliva, closely simulating the actual oral microbial environment [30]. Thus, the current study provides more clinically relevant insights into the efficacy of DC-PL.

Similarly, aciduric bacterial counts indicated that DC-PL exhibited antimicrobial activity comparable to that of PD (Table 2). Aciduric bacteria such as *Streptococcus mutans* and *Lactobacillus* metabolize carbohydrates into lactic acid, thereby reducing the pH of the oral environment [31]. This acidification promotes the overgrowth of acid-resistant pathogenic bacteria while suppressing the proliferation of commensal microbes, potentially leading to microbial dysbiosis [32]. In the present study, DC-PL effectively inhibited aciduric bacteria, thereby preventing the excessive acidification of the oral environment. These findings suggest that DC-PL may contribute to the preservation of the balance of the oral microbial ecosystem, a crucial factor in maintaining oral health.

While chemical denture cleansers are known for their potent bactericidal effects, they have also been associated with material degradation [33,34], mucosal irritation, and even allergic reactions [35]. Most previous studies have primarily focused on assessing the impact of denture cleansers on material stability and potential degradation [36,37,38], with relatively little attention given to their effects on intraoral soft tissues, particularly the oral mucosa. Consequently, comprehensive evaluations of their biocompatibility remain limited. Given that the oral mucosa is the primary tissue in direct contact with denture cleansers, the biocompatibility of cleansers is a key criterion for evaluating the clinical safety of such products [39]. To address this gap, the authors of present study examined an in vivo biocompatibility assessment and confirmed that DC-PL poses a minimal risk to oral mucosal health. Specifically, DC-PL demonstrated an oral mucosal irritation index of 0, indicating no adverse effects on the oral mucosa (Table 3). Histological analysis further confirmed these results, with no epithelial alterations, leukocyte infiltration, vascular congestion, or edema observed in the experimental group (Figure 4).

Nonetheless, this study has some limitations. First, the oral microcosm biofilms were developed using saliva obtained from a single donor. Given that the composition of oral microbiota varies among individuals [40], this could influence biofilm characteristics. However, previous studies suggest that ecological factors play a more significant role in shaping biofilm pathogenicity than individual microbial differences [41]. In addition, when environmental conditions are standardized, biofilms derived from either single or multiple donors tend to exhibit comparable cariogenic potential [42,43]. Therefore, use of saliva from a single donor was deemed sufficient for evaluating biofilm responses to environmental changes. Second, this study did not involve a detailed microbiome analysis of the saliva sample. While interindividual differences in microbial composition exist, the primary objective of this study was to assess the effects of DC-PL on ecological dynamics within oral biofilms. Incorporating microbial profiling in future studies could provide deeper insights into the specific compositional shifts induced by DC-PL, as well as its broader impact on microbial communities. Third, this study did not assess the selective effects of DC-PL on microbial populations associated with oral health. While bacterial viability and aciduric bacterial counts were analyzed as key indicators, further investigation is needed to determine the interaction between DC-PL and both beneficial and pathogenic microorganisms. Understanding its role in modulating microbial balance could help clarify its potential as a therapeutic agent for oral health maintenance. Fourth, although this study demonstrated that DC-PL effectively inhibited biofilm maturation, it did not examine the specific mechanisms by which its active compounds, such as polyphenols and flavonoids, influence biofilm structure and composition. Future studies should employ advanced analytical techniques such as metagenomic and proteomic profiling to elucidate the precise biochemical interactions between DC-PL and biofilm communities. Such an approach would provide a more comprehensive understanding of how DC-PL targets pathogenic biofilms while preserving beneficial microbial populations. Fifth, clinical factors including patient-reported outcomes, user compliance, and subjective evaluations (e.g., taste, odor, and ease of use) were not explored in this study. Future clinical trials incorporating these parameters would help confirm the practical usability, patient acceptability, and the overall clinical value of DC-PL in real-world settings. Finally, the treatment duration for PD was set at 1 min, which is shorter than the manufacturer’s recommended soaking time of 5 min. This decision was made to ensure consistency across all treatment groups and to specifically assess the antimicrobial efficacy of DC-PL within a clinically relevant short exposure time. However, the shortened exposure time may have prevented PD from fully exhibiting its antimicrobial potential. Previous studies have shown that PD achieves optimal efficacy when applied for at least 5 min [29,44]. Because PD is a well-established chemical denture cleanser with documented effectiveness, a longer exposure time might have enhanced its antimicrobial activity, potentially influencing the comparative outcomes observed in this study. Future studies should extend the exposure duration for both PD and DC-PL to determine whether prolonged treatment enhances their antimicrobial efficacy and to better evaluate their comparative effectiveness over time.

Despite these limitations, to the best of our knowledge, this study is the first to evaluate the antimicrobial efficacy and biocompatibility of DC-PL using an oral microcosm biofilm model that reflects the complexity of the oral ecosystem. Most previous studies have relied on single-species biofilm models or focused on specific pathogens [45,46,47]. However, the ecological plaque hypothesis suggests that oral diseases arise from microbial dysbiosis driven by environmental factors [48], not merely from the overgrowth of particular pathogens [41]. Furthermore, earlier research on DC-PL primarily examined its effects on the physical properties of denture materials, such as surface roughness and microhardness [36]. In contrast, this study not only demonstrated that DC-PL effectively suppresses biofilm pathogenicity but also confirmed its biocompatibility, reinforcing its safety and potential for intraoral application. These findings highlight the promising potential of ecological modalities like DC-PL to advance the prevention and management of oral diseases.

## 5. Conclusions

This study demonstrated that DC-PL effectively inhibited the growth of pathogenic bacteria in oral microcosm biofilms, showing antimicrobial efficacy comparable with that of PD. In addition, it exhibited notable inhibitory activity against aciduric bacteria, suggesting its potential benefits in maintaining oral microbiome balance and preventing microbial dysbiosis. Importantly, DC-PL also demonstrated excellent biocompatibility, causing no irritation or adverse histopathological effects on oral mucosal tissues. These findings suggest that DC-PL has the potential to serve as a clinically safe and effective natural denture cleanser, offering a viable alternative to conventional chemical cleansers. Nevertheless, further clinical studies involving larger, diverse patient populations and prolonged follow-up periods are necessary to confirm its long-term antimicrobial efficacy, stability, and safety. Such comprehensive evaluations would further substantiate the clinical utility of DC-PL and support its wider adoption in clinical practice.

## Figures and Tables

**Table 1:** Changes in the red fluorescence intensity (Ratio_R/G_) of oral microcosm biofilms after treatment with different agents.

Treatment	n	Ratio_R/G_	
		Baseline	After treatment
Distilled water	14	1.33 (0.07) ^a^	1.28 (0.07) ^a^
Denture cleanser containing *Paeonia lactiflora* extract	14	1.30 (0.03) ^a^	1.21 (0.06) ^b^
Polident^®^	14	1.32 (0.07) ^a^	1.23 (0.07) ^b^
*p*-values ^†^		0.407	0.026

All data are presented as mean (standard deviation). ^†^ *p*-values were obtained using one-way analysis of variance. ^a,b^ Different letters within the same column indicate significant between-group differences according to Tukey’s post hoc test (α = 0.05). Ratio_R/G_, ratio of red to green fluorescence intensity.

**Table 2:** Aciduric bacterial counts after treatment.

Treatment	n	logCFU/mL
Distilled water	14	5.98 (0.33) ^a^
Denture cleanser containing *Paeonia lactiflora* extract	14	5.50 (0.65) ^b^
Polident^®^	14	5.73 (0.46) ^b^
*p*-value ^†^		0.048

All data are presented as mean (standard deviation). ^†^ *p*-value was obtained using one-way analysis of variance. ^a,b^ Different letters within the same column indicate significant between-group differences according to Tukey’s post hoc test (α = 0.05). CFU, colony-forming unit.

**Table 3:** Histopathological evaluation and oral mucosal irritation index.

Reaction	Control			DC-PL		
	Hamster 1	Hamster 2	Hamster 3	Hamster 1	Hamster 2	Hamster 3
Epithelium	0	0	0	0	0	0
Leucocyte infiltration	1	1	1	1	1	1
Vascular congestion	0	0	0	0	0	0
Edema	0	1	1	1	0	0
Total	1	2	2	2	1	1
Mean	1.7			1.3		
Irritation index	0					

The mean value was calculated as the total score divided by the number of observations. The oral mucosal irritation index was calculated as the difference between the mean value for the control group and that for the DC-PL group. DC-PL, denture cleanser containing *Paeonia lactiflora* extract.

## Data Availability

The datasets analyzed in this study can be obtained upon request from the corresponding author. Due to intellectual property rights restrictions, data are not publicly accessible.

## Abbreviations

The following abbreviations are used in this manuscript:

DC-PL	denture cleanser containing *Paeonia lactiflora* extract
DW	distilled water
Ratio_R/G_	ratio of red to green fluorescence intensity
PL	*Paeonia lactiflora*
ANOVA	analysis of variance
PBS	phosphate-buffered saline
